# Moderate dietary boron supplementation improved growth performance, crude protein digestibility and diarrhea index in weaner pigs regardless of the sanitary condition

**DOI:** 10.5713/ab.21.0110

**Published:** 2021-06-24

**Authors:** Hyun Min Cho, Shemil Priyan Macelline, Samiru Sudharaka Wickramasuriya, Taeg Kyun Shin, Eunjoo Kim, Hong Cheol Son, Jung Min Heo

**Affiliations:** 1 Department of Animal Science and Biotechnology, Chungnam National University, Daejeon 34134, Korea; 2 School of Life and Environmental Sciences, The University of Sydney, NSW 2006, Australia

**Keywords:** Boron, Diarrhea Index, Pigs, Protein Digestibility

## Abstract

**Objective:**

The study was conducted to investigate the impact of boron supplementation on nutrient digestibility, inflammatory responses, blood metabolites and diarrhea index, and their relevance to growth performance in weaned pigs housed in good and poor sanitary environments for 14 days after weaning.

**Methods:**

A total of 108 male pigs (Duroc×[Yorkshire×Landrace]) weaned at 21 days of age were used in a randomized complete block design with 2×3 factorial arrangement. Pigs were assigned to three boron treatments (0, 5, and 10 mg/kg) under two environments (good and poor sanitary) to give six replicates per treatment (3 pigs per replicate). On 0, 7, and 14 days, one pig per replicate was euthanized to collect, ileum tissue samples, and rectal fecal samples.

**Results:**

Boron supplementation quadratically influenced (p<0.001) feed intake and weight gain in pigs housed in good sanitary conditions from 1 to 14 days post-weaning where pigs offered 5 mg/kg boron optimized weight gain and feed intake. There is a quadratic interaction (p = 0.019) on feed intake for 1 to 14 days post-weaning where 5 mg/kg boron increased feed intake in good sanitary conditions. Pigs housed in the poor sanitary environment decreased (p<0.001) villus height and crypt depth in ileum at days 7 and 14. On day 7 and 14, crude protein digestibility was quadratically influenced (p<0.05) by boron supplementation. Boron supplementation linearly increased (p<0.05) plasma calcium and cholesterol levels whilst linearly (p = 0.005) reducing plasma triglyceride concentrations. Diarrhea index was quadratically influenced (p<0.05) by boron supplementations regardless of sanitary conditions where 5 mg/kg boron inclusion achieved the lowest diarrhea index.

**Conclusion:**

Pigs offered 5 mg/kg of boron increased weight gain which may be deduced by improved dry matter, crude protein, and energy digestibility regardless of the sanitary conditions.

## INTRODUCTION

Boron has been identified as an important element in animals because of its impact on metabolic responses, energy utilization and homeostatic mechanisms in animal tissues [1]. It has been found that boron supplementation improved the growth performance in pigs may be due to its metabolic functions and positive impact on nutrient absorption in the small intestine as described by Armstrong et al [2,3], respectively. Moreover, the beneficial impact of dietary boron on bone strength was discussed in Chen et al [4] such that boron is an indispensable mineral in bone development, cell proliferation and mineralization in bone tissues. Moreover, it has been stated that boron-supplemented diets have increased bone yield stress and bending moment in pigs [5]. Armstrong et al [2] demonstrated that a diet supplemented with 5 ppm boron improved feed conversation efficiency in pigs. Subsequent experiments [4–6] revealed that pigs fed boron-supplemented diets showed higher growth rates, increased plasma mineral concentrations, and serum metabolite concentrations than pigs fed diets without boron. However, high boron contents in diets adversely impact growth performance and immune organs (e.g., thymus, spleen, and bursa) in animals which is dependable on the type of animal and their body weights (BWs) [7].

Pigs housed in facilities with poor sanitary conditions exhibit an increased incidence of inflammatory disease that leads to poor growth performance [8]. Proinflammatory cytokines play a major role in the animal immune system under inflammatory disease conditions by stimulating the possible defensive mechanism and inflammatory responses [9]. Supplementation of antimicrobial growth promoters (AGP) has long been used to overcome enteric challenges but presently there are many limitations for AGP usage for the pig industry mainly due to antimicrobial resistance and environmental pollution [10]. Interestingly it has been found that boron has the ability to improve nutrient digestibility by moderating nutrient transporters and stimulate the immune system by producing pro-inflammatory cytokines [1–6]. Therefore, boron may be a suitable candidate for replacing AGP in pigs. Testing the impact of boron for pigs under poor sanitary conditions may help to understand its beneficial influences in challenging conditions. In previous studies [2,3,5,6], pigs were exposed to 5 to 15 mg/kg of dietary boron. However, as a safe margin, 5 and 10 mg/kg levels of boron were tested in the present study since a higher concentration of dietary boron may cause toxicity in animals [7]. Consequently, this study was conducted to test the hypothesis that dietary boron supplementation improves growth performance and nutrient digestibility with improved inflammation responses and diarrhea index values in pigs housed in an environment with poor sanitary conditions for 14 days post-weaning.

## MATERIALS AND METHODS

The experimental protocol used in this study was approved by the Animal Ethics Committee of the Chungnam National University (CNU-00485).

### Experimental design, animals, housing and diets

This experiment was conducted as a 2×3 factorial treatment arrangement, with respective factors being i) two environmental conditions (good sanitary, cleaned and disinfected previously unpopulated rooms; poor sanitary, manure application with no cleaning or disinfection of a previously populated room) and ii) three levels of boron (0, 5, and 10 mg/kg).

A total of 108 male pigs (Duroc×[Yorkshire×Landrace]) weaned at 21 days of age with initial BW of 6.59±1.86 kg were used. Pigs were obtained from a local government experimental farm (Chungnam Livestock Research Institute, Cheongyang, Korea) at weaning and transported to an animal research farm. The facility contained two rooms that allowed pigs to be housed separately in good sanitary or poor sanitary environments, to avoid any cross contamination. Pigs were allocated to their experimental treatments based on initial BW for blocking the factors within two rooms (randomized complete block design). Six replicate pens with three pigs per pen were allocated to each treatment. Each pen was equipped with a nipple bowl drinker and a metal trough. The ambient temperature was maintained at 29°C± 1°C for the initial week and then gradually decreased by 1°C every week. Pigs were offered the experimental diets on an *ad libitum* basis for 2 weeks and were always freely accessible to the freshwater.

Experimental diets were formulated to have similar crude protein and gross energy levels and meet or exceed the NRC [11] recommendations (Table 1). Three levels of boron (0, 5, and 10 mg/kg) were included by supplementing boric acid (Junsei Chemical Co., Ltd., Tokyo, Japan) by top dressing. Chromic oxide was added to experimental diets (0.3%) as an indigestible marker for the calculation of apparent total tract digestibility (ATTD) of dry matter (DM), crude protein, and energy.

### Measurement of BW and feed consumption

Individual BW was measured at days 0, 7, and 14 of the experiment. Feed intake of each pen was recorded on days 7 and 14 as feed disappearance from the feeder. Based on the measurements, the average daily gain (ADG, g/pig/d), average daily feed intake (ADFI, g/pig/d) and gain-to-feed (G:F) ratio were calculated.

### Assessment of fecal consistency and the incidence of diarrhea

Two weeks after weaning, feces were visually assessed daily at 1000 h to determine fecal consistency scores and the incidence of diarrhea using the procedure described by Heo et al [12] during the experimental period (0 to 14 days). Feces were assessed using the fecal consistency score according to Marquardt et al [13] using a subjective score on a 3-point scale ranging from 1 to 3: 1, well formed; 2, sloppy; 3, diarrhea. On days 7 and 14, fecal samples were collected from the rectum and stored in a labeled sterile container and kept frozen at −20°C until analyses for DM, crude protein, and gross energy.

### Post-mortem procedure

One pig per pen was euthanized on days 0, 7, and 14 according to modified procedures described in [13] to collect blood and intestinal tissue samples. Pigs were administered a single intramuscular injection of 0.1 mL SUCCIPHARM (50 mg suxamethonium chloride, Komipharm International Co., Ltd., Shieung, Korea) to induce general anesthesia, and then blood samples (5 to 10 mL) were collected via jugular vein puncture using vacuum tubes coated with lithium heparin (Vacutainer; Becton Dickinson, Franklin Lakes, NJ, USA). The blood plasma was separated by centrifugation (Micro 12; Hanil Science Co., Ltd., Incheon, Korea) at 2,000×g for 10 min at 4°C and stored at −80°C until analysis. Thereafter, pigs were introduced into a chamber where residual air was rapidly flushed with 100% CO_2_ until death was confirmed. The abdominal cavity was exposed by midline laparotomy. For measurement of villous height and crypt depth, 3 to 4 cm section segment of the small intestine was removed at the ileum (approximately 5 cm cranial to the ileo-caecal junction) as described by Heo et al [14] and carefully washed with phosphate buffered saline and preserved in 10% formalin solution for subsequent histological examination.

### Mucosal histology

Tissue preparation and methods for microscopic measurements were conducted following standard histological procedures described by Heo et al [14]. After fixation in the 10% phosphate buffered saline for several days, ileum sections were excised, dehydrated, and embedded in paraffin wax. From each of the embedded samples, 6 transverse sections (4 to 6 μm) were cut, stained with hematoxylin and eosin, and mounted on glass slides. The height of 10 well oriented villi and their associated crypts were measured with a light microscope (OLYMPUS CX31, Tokyo, Japan) using a calibrated eyepiece graticule [15].

### Chemical analyses

Diet DM concentration was determined according to the AOAC 930.15 [16] by oven drying 5 g of samples at 135°C for 2 h. The gross energy content of experimental diets and feces were measured using an isoperibol bomb calorimeter (model 6300; Parr Instrument, Moline, IL, USA) which has been calibrated using benzoic acid as a standard. Nitrogen contents in the experimental diets and feces were determined by the combustion method of AOAC 990.03 [16] using the LECO nitrogen analyzer (model CNS-2000; LECO Corp., St. Joseph, MI, USA) and crude protein was calculated as N×6.25.

Plasma samples were used to quantify the concentrations of proinflammatory cytokines by using commercially available ELISA kits (R&D Systems, Minneapolis, MN, USA) for interleukin 1β (IL-1β) and tumor necrosis factor-α (TNF-α) according to the manufacturer’s instructions following the method described by Piñeiro et al [17].

### Digestibility calculation

The ATTD of DM, crude protein, and gross energy were calculated for each diet according to Stein et al [18] by using the following equation.


ATTD (%)=1-(Nutrient in feces/Nutrient diet)×(Cr in diet/Cr in feces)×100%

### Statistical analyses

The pen was the experimental unit for all responses. Data were analyzed as randomized complete block design using the general linear model procedure of SPSS software (Version 22; IBM SPSS 2013). Orthogonal polynomial contrast was performed to get the linear and quadratic effect of dietary boron and interaction effects on response parameters. Pigs were blocked based on weaning weight and the block was used as a random factor in the model for all measured experimental variables. Body weight, ileum morphology and immune response on day 0 were included in the model as a covariate for analyses of growth performance, ileal architecture and cytokine responses. Data for the incidence of post-weaning diarrhea were expressed as the mean percentage of days with diarrhea relative to the 14 days after weaning [12].

## RESULTS

### Growth performance

The effects of dietary boron and environmental condition on growth performance in weaned pigs were presented in Table 2. There was no mortality found through the experiment period. There was a quadratic interaction on ADFI for 1 to 7 (p = 0.009) and 1 to 14 (p = 0.019) days where pigs supplemented 5 mg/kg attained the highest feed intake under good sanitary conditions but not in the poor sanitary conditions. During 8 to 14 days, a linear interaction effect was found on G:F ratio where the transition of dietary boron from 0 to 10 mg/kg linearly reduced G:F ratio (p = 0.049). Boron supplementation quadratically influenced ADG (p< 0.001) and G:F (p = 0.014) in pigs from 1 to 14 days post-weaning regardless of sanitary conditions.

### Intestinal morphology

The impact of environmental conditions and dietary boron levels on ileal morphology in weaned pigs was tabulated in Table 3. There was no interaction effect found on ileal morphology during the experimental period. Pigs housed in good sanitary conditions obtained higher villus height, crypt depth and lower villous height to crypt depth ratio (V:C) than their counterpart (p<0.01).

### Apparent digestibility of nutrients

Effects of environmental condition and dietary boron levels on ATTD of DM, crude protein, and energy in weaned pigs shown in Table 4. On day 7, the interaction effect indicates that crude protein digestibility linearly reduced by supplemented boron in good sanitary conditions (p = 0.013). Good sanitary conditions improved DM, crude protein, and energy digestibility compared to poor sanitary conditions (p<0.05). Boron supplementation quadratically influenced DM, crude protein, and energy digestibility at day 7 and 14 (p<0.05).

### Pro-inflammatory cytokines

Table 5 showed the effect of environmental conditions and boron levels on pro-inflammatory cytokines in the gut mucosa of weaned pigs on day 7 and 14. Neither interaction nor main treatment effect was found on pro-inflammatory cytokine levels in the present study.

### Plasma composition

Plasma concentrations of calcium, phosphorous, cholesterol and triglyceride at day 7- and 14-days post-weaning were presented in Table 6. There were linear (p = 0.014) and quadratic (p = 0.031) interactions obtained on plasma cholesterol concentrations on day 7. Sanitary conditions influenced plasma phosphorus levels on day 7 and 14 (p<0.001). On day 7, supplemented boron linearly increased plasma calcium levels (p = 0.026) but linearly reduced phosphorous (p<0.001) and triglyceride (p<0.001) levels. On day 14, supplemented boron linearly increased plasma calcium (p = 0.004) and cholesterol levels (p = 0.037) whereas reduced triglyceride levels (p<0.005).

### Diarrhea index

Impacts of environmental conditions and supplemented boron levels on diarrhea index in weaned pigs are tabulated in Table 7. No interaction effect was observed (p>0.05); otherwise, prominent environment effect obtained for up to 7 days post-weaning where pigs housed in the poor sanitary conditions obtained higher (p = 0.05) diarrhea index than their counterpart. Supplemented boron quadratically influenced diarrhea index for 1 to 7 days (p = 0.022), 8 to 14 days (p = 0.020), and 1 to 14 days (p = 0.002) where lower diarrhea index was supported by the pigs offered 5 mg/kg supplemented diets.

## DISCUSSION

It has been found that pigs fed diets with 5 mg/kg boron supplementation improved growth performance in pigs under conventional farm conditions [2]. In the present study, the quadratic effects of boron supplementations on growth performance indicative that 5 mg/kg boron supplementation supported higher feed intake, weight gain, and G:F ratio in weaner pigs. However, the transition of boron supplementation from 0 to 10 mg/kg reduced feed intake by 35% (442 vs 289 g/pig/d, p = 0.006) and 35% (380 vs 245 g/pig/d, p = 0.022) in poor sanitary and good sanitary conditions, respectively, where the significance of pair-wise comparisons are shown in parentheses for 1 to 14 days post-wean. It has been reported that dietary boron can be toxic to animals by damaging immune organs; consequently, decreased feed intake and reduced weight gain [7]. Therefore, 10 mg/kg of boron supplementation may have become toxic regardless of sanitary conditions for weaner pigs in the present study. Consequently, boron supplementation at 5 mg/kg may be the safe margin in weaner pigs which is needed to optimize their growth performance. However, the quadratic equation obtained for weight gain (Y_ADG_ = 363+42.8X_(boron)_− 5.76X^2^_(boron)_; r = 0.632, p<0.001) showed that 3.71 mg/kg level of boron supplementation attained the highest ADG of 442 g/pig/d for 1 to 14 days post-wean in the present study.

It has been reported that boric acid supplementation with water (640 mg/L) stimulates intestinal cell apoptosis in poultry and resulted from higher intestinal cell proliferation as a compensative reaction [7]. Moreover, intestinal cell apoptosis contributed to cell proliferation could results lower V:C ratio due to higher demand for intestinal cell turnover to maintain the villus physiology. However, it was unable to detect such an impact in intestinal morphology from dietary boron supplementation in the present study. It has been reported that Pigs reared under unclean sanitary conditions had shorter villus height and less crypt depth in Zhao et al [19] which is similar to the present study. Reduced villus height and crypt depth were resulted due to the higher pathogenic bacteria activity and their products in the gastrointestinal tract as reported by Ao et al [20]. Pigs in poor sanitary conditions have a higher possibility to contain a large number of pathogenic bacteria in their gastrointestinal tract and it could be the reason for impaired gut morphology obtained in the present study. However, impaired intestinal morphology was not reflected in growth performance in the present study. The possible explanation for these outcomes may be due to the reporting morphology of ileum instead of jejunum, thus jejunum is the major site where the majority of nutrient absorption takes place whilst most of the bacterial activity takes place in the ileum.

The quadratic effect of dietary boron supplementation indicated that 5 mg/kg boron improved crude protein digestibility on day 7 and 14. Goldbach et al [21] revealed that boron can bind with protease enzyme, which may reduce protein digestibility in animals. Consequently, extensive protease inactivation may be the reason for low crude protein digestibility in pigs offered 10 mg/kg boron diets in the present study. Therefore, low weight gain and G:F ratio in pigs offered 10 mg/kg may be also influenced by poor crude protein digestibility. Suiryanrayna and Ramana [22] reported that lowering gastric pH using weak acids increased protein utilization in pigs by converting inactive pepsinogen to pepsin. In the present study, boron was supplemented as boric acid which is known as weak acid with a pKa value of 9.2 [23] and it also helps to reduce the acid binding capacity in feed. Therefore, the negative impact of boron on protease enzymes may be overcome by the acidity of boric acid when supplementary boron concentration is low (5 mg/kg).

Boron impact on the production of pro-inflammatory cytokinin in pigs is variable in previous studies [3,6]. However, Armstrong and Spears [6] stated that pigs offered boron supplemented diets increased the production of TNF-α under lipopolysaccharide challenge conditions and the mechanism is retained unclear. Pro-inflammatory cytokinin production is important for stimulating the immune system to take place necessary immune responses to protect the body from antigens. Lipopolysaccharides are a type of endotoxins were existing in the membranes of gram-negative bacteria. Poor sanitary conditions influenced pathogenic bacteria activity in the gastrointestinal tract in pigs may reasoning endotoxin leakage to systemic plasma. Therefore, in the present study, it was predicted that dietary boron may influence pro-inflammatory cytokinin production in pigs housed in poor sanitary conditions. However, the outcomes of the present study did not support this hypothesis because both dietary boron and sanitary conditions did not influence the pro-inflammatory cytokinin levels in systemic plasma. Furthermore, it is important to measure plasma endotoxin levels to confirm the endotoxin leakage from the gastrointestinal tract in future studies.

It has been found that boron can influence the utilization and metabolism of calcium, phosphorous, cholesterol, and triglycerides in both humans and animals [7]. In the present study, dietary boron supplementation linearly elevated plasma calcium and phosphorus levels at day 7 and a similar pattern was observed on day 14. Increasing plasma calcium levels may be indicative of improved calcium absorption in the gastrointestinal tract. The evidence for improved calcium absorption with boron supplementation has been reported in Bhasker et al [24] and boron influence on modification of calcium transporters across plasma membranes was reported in Green [25]. As a confounding factor, the acidity of the boric acid used in the present study may be also influenced the calcium and phosphorus absorption due to the fact that increases the solubility of phytic acid under low pH values. However, it is contradictory to the findings in the present study because boron supplementation linearly reduced the plasma phosphorus concentrations. Moreover, the impact of boron supplementation on phosphorus digestibility and plasma concentrations is inconsistent in the previous studies as reported by Mızrak et al [26]. Nevertheless, it has been found that increased boron supplementation to pigs increased phosphorous level in bone whereas calcium level was not impacted [2]. Therefore, boron may be efficiently involving in the deposition of phosphorous in bone from plasma rather than calcium. This concept was also suggested in Green [25].

The impact of boron supplementation on plasma cholesterol and triglycerides in pigs was reported in previous studies [2,3]. The outcomes of the present study are lining with the previous studies where boron supplementation increase plasma cholesterol level [2] and reduced plasma triglyceride levels [27]. However, contradictory results were reported in Armstrong et al [28] such that boron supplementation increased plasma triglyceride levels. It has been reported that boron impact on cholesterol is indirect because boron impact on plasma thyroxine level regulates the cholesterol level as thyroxin has an inverse relationship with plasma cholesterol [29, 30]. The mechanism behind the impact of boron on plasma triglyceride levels remains unclear and further researches needed to confirm suitable explanations. In the small intestine, fats in vegetable oil hydrolyzed to monoglycerides and free fatty acids and absorbed into epithelial cells and resynthesized to triglyceride before entering the systemic plasma [31]. Therefore, reduction of feed intake may be a possible reason for low plasma triglyceride levels in pigs offered 10 mg/kg boron diets.

Diarrhea in pigs mainly caused by the growth of putrefactive microorganisms in the hindgut that is accelerated by the accumulation of undigested protein. The quadratic effect of boron supplementation indicates that 5 mg/kg decreases the diarrhea index in the present study regardless of sanitary conditions for 1 to 14 days. Therefore, the reduction of incidence of diarrhea in pigs offered 5 mg/kg may be caused by improved crude protein digestibility in the present study.

## CONCLUSION

The effect of boron supplementation as a form of boric acid on ADG and G:F ratio in pigs independent to the sanitary conditions such that 5 mg/kg of boron supplementation supported optimum ADG and G:F whereas ADFI was dependent on sanitary conations for 1 to 14 days post-wean. The advantageous effect of 5 mg/kg boron on crude protein, energy and DM digestibility may be the reasons for improved growth performance and low diarrhea index in weaner pigs. Furthermore, poor nutrient digestibility, low plasma triglyceride levels and reduced feed intake reflected the supplementation of 10 mg/kg boron to diets may cause a nutrient deficiency in weaner pigs.

## Figures and Tables

**Table 1:** Ingredient and nutrient composition of the experimental diet (as-fed basis)

Item	
Ingredients (%)	
Corn	34.89
Wheat	12.00
Soybean meal, 44%	28.00
Fish meal	5.00
Dried whey	15.00
Vegetable oil	3.10
Limestone	0.80
Monocalcium phosphate	0.36
Vitamin-mineral premix^1)^	0.40
L-Lys-HCl, 78.8%	0.28
DL-Methionine	0.11
Threonine	0.06
Calculated chemical composition	
Metabolizable energy (kcal/kg)	3,400
Calcium (%)	0.85
Methionine+cysteine (%)	0.84
Lysine (%)	1.50
Analyzed composition	
Gross energy (kcal/kg)	3,900
Crude protein (%)	22.32

1) Provided the following nutrients (per kg of air-dry diet): vitamin A, 7,000 IU; vitamin D_3_, 1,400 IU; vitamin E 20, mg; vitamin K, 1 mg; vitamin B_1_ (thiamine), 1 mg; vitamin B_2_ (riboflavin), 3 mg; vitamin B_6_ (pyridoxine), 1.5 mg; vitamin B_12_ (cobalamin), 15 μg; calcium pantothenate, 10.7 mg; folic acid, 0.2 mg; niacin, 12 mg; biotin, 30 μg; Co, 0.2 mg (as cobalt sulphate); Cu, 10 mg (as copper sulphate); iodine, 0.5 mg (as potassium iodine); iron, 60 mg (as ferrous sulphate); Mn, 40 mg (as manganous oxide); Se, 0.3 mg (as sodium selenite); Zn, 100 mg (as zinc oxide).

**Table 2:** Effects of environment and boron levels (mg/kg) on average daily gain (ADG, g/pig/d), average feed intake (ADFI, g/pig/d) and gain-to-feed ratio (G:F, g/g) in weaned pigs from day 1 to 14 post-weaning

Environment	Boron levels	Day 1–7			Day 8–14			Day 1–14		
		
		ADFI	ADG	G:F	ADFI	ADG	G:F	ADFI	ADG	G:F
Good sanitary	0	230	158	0.69	530	545	1.03	380	351	0.93
	5	368	275	0.74	652	660	1.01	510	467	0.91
	10	178	182	1.01	312	203	0.60	245	192	0.74
Poor sanitary	0	271	195	0.69	613	548	0.86	442	371	0.83
	5	233	152	0.46	556	661	1.17	394	407	0.99
	10	180	174	1.05	398	299	0.78	289	237	0.87
SEM		32.2	41.0	0.134	54.6	67.4	0.083	39.1	44.5	0.056
		--------------------------------------------------- Mean value of main effects ----------------------------------------------------------								
Environment										
Good sanitary		258	205	0.86	500	473	0.88	379	342	0.87
Poor sanitary		228	174	0.70	521	500	0.94	375	334	0.89
Boron levels										
0		250	176	0.73	573	549	0.95	412	366	0.89
5		300	214	0.63	605	662	1.09	452	439	0.96
10		179	178	0.99	353	248	0.69	266	210	0.79
		------------------------------------------------------ Significance of main effects ----------------------------------------------------------								
Environment		0.275	0.086	0.078	0.652	0.637	0.434	0.895	0.831	0.694
Boron supplementation										
Linear		0.036	0.813	0.016	<0.001	<0.001	0.004	0.001	0.001	0.078
Quadratic		0.005	0.245	0.016	0.006	<0.001	0.001	0.002	<0.001	0.014
		------------------------------------ Significance of interaction effects from orthogonal contrast ----------------------------								
Linear		0.538	0.570	0.868	0.976	0.486	0.049	0.817	0.781	0.051
Quadratic		0.009	0.051	0.205	0.067	0.686	0.268	0.019	0.245	0.537

Each simple effect mean represents 6 replicate pens.

SEM, standered error of mean.

**Table 3:** Effects of environment and boron levels (mg/kg) on ileal morphology in weaned pigs on day 7 and 14

Environment	Boron levels	Day 7			Day 14		
	
		Villus height (V, μm)	Crypt depth (C, μm)	V:C ratio	Villus height (V, μm)	Crypt depth (C, μm)	V:C ratio
Good sanitary	0	616	247	2.51	736	319	2.38
	5	611	272	2.27	699	288	2.51
	10	588	265	2.16	798	340	2.37
Poor sanitary	0	234	85	2.82	286	50	4.77
	5	181	75	2.42	266	53	3.43
	10	209	84	2.61	281	31	3.33
SEM		18.1	10.0	0.173	32.5	12.8	0.456
		------------------------------------------------------ Mean value of main effects ------------------------------------------------------					
Environment							
Good sanitary		625	250	2.26	763	309	2.49
Poor sanitary		188	97	2.67	259	87	3.78
Boron levels							
0		424	167	2.65	510	196	3.60
5		400	173	2.37	486	183	2.95
10		396	180	2.39	537	214	2.84
		------------------------------------------------------ Significance of main effects -------------------------------------------------------					
Sanitary condition		<0.001	<0.001	0.008	<0.001	<0.001	0.002
Boron							
Linear		0.130	0.186	0.126	0.422	0.187	0.106
Quadratic		0.528	0.885	0.307	0.206	0.055	0.496
		------------------------------- Significance of interaction effects from orthogonal contrast -----------------------------					
Linear		0.938	0.138	0.687	0.310	0.848	0.122
Quadratic		0.124	0.286	0.437	0.376	0.074	0.351

Each simple effect mean represents 6 replicate pens.

SEM, standered error of mean.

**Table 4:** Effects of environment and boron levels (mg/kg) on apparent total tract digestibility (%) of nutrients in weaned pigs on days 7 and 14

Environment	Boron levels	Day 7			Day 14		
	
		Dry matter	Crude protein	Energy	Dry matter	Crude protein	Energy
Good sanitary	0	92.4	89.6	90.2	92.9	88.3	91.6
	5	94.6	91.8	93.9	93.2	91.7	92.5
	10	88.6	80.5	86.8	91.0	85.4	90.3
Poor sanitary	0	88.9	75.4	87.2	87.4	79.2	84.9
	5	91.5	86.8	90.3	92.7	89.8	91.6
	10	82.3	74.9	81.4	87.5	80.6	86.7
SEM		0.83	1.73	0.91	1.63	2.88	1.33
		--------------------------------------------------- Mean value of main effects ---------------------------------------------------------					
Environment							
Good sanitary		91.9	87.3	90.3	92.4	88.4	91.4
Poor sanitary		87.6	79.0	86.3	89.2	83.3	87.58
Boron levels							
0		90.6	82.5	88.7	90.1	83.6	88.1
5		93.1	89.3	92.1	93.0	90.9	92.1
10		85.4	777	84.1	89.3	83.0	88.5
		----------------------------------------------------------- Significance of main effects -------------------------------------------------					
Environment		0.001	<0.001	<0.001	0.018	0.024	0.025
Boron supplementations							
Linear		<0.001	0.010	<0.001	0.595	0.840	0.834
Quadratic		<0.001	<0.001	<0.001	0.018	0.002	0.029
		---------------------------- Significance of interaction effects from orthogonal contrast ---------------------------------					
Linear		0.771	0.013	0.723	0.132	0.230	0.149
Quadratic		0.051	0.205	0.190	0.869	0.888	0.968

Each simple effect mean represents 6 replicate pens.

SEM, standered error of mean.

**Table 5:** Effects of environment and boron levels (mg/kg) on pro-inflammatory cytokines of gut mucosa in weaned pigs on days 7 and 14

Environment	Boron levels	Day 7		Day 14	
	
		IL-1β (pg/mL)	TNF-α (pg/mL)	IL-1β (pg/mL)	TNF-α (pg/mL)
Good sanitary	0	23.6	1.2	65.5	1.8
	5	9.6	3.3	11.4	1.7
	10	24.6	0.7	56.3	1.2
Poor sanitary	0	14.1	31.7	15.7	2.3
	5	69.0	2.6	44.7	1.9
	10	43.1	1.6	26.8	2.9
SEM		27.11	16.93	31.16	1.13
		---------------------------------------------------- Mean value of main effects ----------------------------------------------			
Environment					
Good sanitary		19.3	1.7	44.4	1.6
Poor sanitary		42.1	11.9	29.1	2.3
Boron levels					
0		18.9	16.4	40.6	2.1
5		32.3	3.0	28.0	1.8
10		33.9	1.1	41.5	2.0
		------------------------------------------------------ Significance of main effects ---------------------------------------------			
Environment		0.319	0.473	0.554	0.413
Boron supplementations					
Linear		0.597	0.379	0.379	0.984
Quadratic		0.581	0.702	0.702	0.812
		----------------------- Significance of interaction effects from orthogonal contrast -----------------------------			
Linear		0.622	0.395	0.749	0.604
Quadratic		0.249	0.588	0.193	0.648

Each simple effect mean represents 6 replicate pens.

IL-1β, interleukin-1β; TNF-α, tumor necrosis factor-α; SEM, standered error of mean.

**Table 6:** Effects of environment and boron levels (mg/kg) on nutrient composition in systemic plasma of weaned pigs on days 7 and 14

Environment	Boron levels	Day 7				Day 14			
	
		Calcium (mg/dL)	Phosphorous (mg/dL)	Cholesterol (mg/dL)	Triglyceride (mg/dL)	Calcium (mg/dL)	Phosphorous (mg/dL)	Cholesterol (mg/dL)	Triglyceride (mg/dL)
Good sanitary	0	10.5	7.4	59.1	78.2	12.2	7.8	68.1	114.3
	5	10.7	7.2	60.8	43.1	12.4	7.2	68.2	79.4
	10	11.0	6.7	62.6	32.6	12.9	7.2	78.3	74.1
Poor sanitary	0	10.2	7.1	79.2	59.1	12.2	10.2	68.9	98.7
	5	10.7	6.2	53.7	49.4	12.7	9.3	68.3	70.7
	10	10.7	5.5	61.1	35.1	13.0	9.0	84.4	65.2
SEM		0.21	0.28	4.23	7.83	0.25	0.48	6.18	12.41
		-------------------------------------------------------------------- Mean value of main effects ------------------------------------------------------							
Environment									
Good sanitary		10.8	7.1	60.9	51.3	12.4	7.4	71.9	89.3
Poor sanitary		10.5	6.3	64.7	47.9	12.6	9.5	73.	78.2
Boron levels									
0		10.4	7.3	69.2	69.0	12.2	9.0	68.0	106.5
5		10.7	6.7	57.3	46.2	12.5	8.3	68.8	75.0
10		10.9	6.1	61.9	33.8	13.0	8.1	81.3	69.7
		-------------------------------------------------------------------- Significance of main effects ------------------------------------------------------							
Environment		0.214	0.001	0.276	0.595	0.338	<0.001	0.741	0.282
Boron supplementations									
Linear		0.026	<0.001	0.091	<0.001	0.004	0.070	0.037	0.005
Quadratic		0.658	0.898	0.030	0.465	0.827	0.486	0.277	0.231
		----------------------------------------- Significance of interaction effects from orthogonal contrast ---------------------------------							
Linear		0.966	0.080	0.014	0.173	0.765	0.612	0.613	0.789
Quadratic		0.560	0.753	0.031	0.287	0.420	0.988	0.722	0.868

Each simple effect mean represents 6 replicate pens.

SEM, standered error of mean.

**Table 7:** Effects of environment and boron levels (mg/kg) on diarrhea index in weaned pigs on days 7 and 14

Environment	Boron levels	Day 1–7	Day 8–14	Day 1–14
Good sanitary	0	3.57	17.88	10.70
	5	0.00	3.58	1.78
	10	3.57	13.10	10.73
Poor sanitary	0	7.15	14.28	10.73
	5	0.00	3.57	1.78
	10	21.43	21.43	21.43
SEM		4.339	6.792	4.071
		--------------------------------------------------- Mean value of main effects ----------------------------------------------		
Environment				
Good sanitary		2.38	13.10	7.73
Poor sanitary		9.52	13.10	11.31
Boron levels				
0		5.36	16.08	10.71
5		0.00	3.57	1.78
10		12.50	19.64	16.08
		------------------------------------------------------- Significance of main effects -------------------------------------------		
Environment		0.050	0.999	0.288
Boron supplementation				
Linear		0.107	0.603	0.195
Quadratic		0.022	0.020	0.002
		------------------------------- Significance of interaction effects from orthogonal contrast --------------------		
Linear		0.107	0.600	0.197
Quadratic		0.161	0.999	0.451

Each simple effect mean represents 6 replicate pens.

SEM, standered error of mean.
